# Time-dependent repolarization changes following left bundle branch area pacing vs. conventional biventricular pacing in patients with dyssynchronous heart failure

**DOI:** 10.1093/europace/euaf034

**Published:** 2025-02-15

**Authors:** Karin C Smits, Edoardo Bressi, Leonard M Rademakers, Jesse H J Rijks, Antonius M W van Stipdonk, Elien B Engels, Aaron Isaacs, Ben J M Hermans, Domenico Grieco, Justin G L M Luermans, Kevin Vernooy, Frits W Prinzen, Uyên Châu Nguyên

**Affiliations:** Department of Physiology, Maastricht University Medical Center+, Cardiovascular Research Institute Maastricht, P.O. Box 616 6200 MD, Universiteitssingel 50, Maastricht 6229 ER, The Netherlands; Department of Cardiology, Policlinico Casilino, Rome 00169, Italy; Department of Cardiology, Catharina Ziekenhuis, Eindhoven 5623 EJ, The Netherlands; Department of Cardiology, Maastricht University Medical Center+, Cardiovascular Research Institute Maastricht, Maastricht 6229 HX, The Netherlands; Department of Cardiology, Maastricht University Medical Center+, Cardiovascular Research Institute Maastricht, Maastricht 6229 HX, The Netherlands; Department of Medical Physics, Catharina Ziekenhuis, Eindhoven 5623 EJ, The Netherlands; Department of Physiology, Maastricht University Medical Center+, Cardiovascular Research Institute Maastricht, P.O. Box 616 6200 MD, Universiteitssingel 50, Maastricht 6229 ER, The Netherlands; Maastricht Centre for Systems Biology, Faculty of Science and Engineering, Maastricht 6229 EN, The Netherlands; Department of Cardiology, Maastricht University Medical Center+, Cardiovascular Research Institute Maastricht, Maastricht 6229 HX, The Netherlands; Department of Cardiology, Policlinico Casilino, Rome 00169, Italy; Department of Cardiology, Maastricht University Medical Center+, Cardiovascular Research Institute Maastricht, Maastricht 6229 HX, The Netherlands; Department of Cardiology, Maastricht University Medical Center+, Cardiovascular Research Institute Maastricht, Maastricht 6229 HX, The Netherlands; Department of Physiology, Maastricht University Medical Center+, Cardiovascular Research Institute Maastricht, P.O. Box 616 6200 MD, Universiteitssingel 50, Maastricht 6229 ER, The Netherlands; Department of Cardiology, Maastricht University Medical Center+, Cardiovascular Research Institute Maastricht, Maastricht 6229 HX, The Netherlands

**Keywords:** Cardiac resynchronization therapy, Biventricular pacing, Left bundle branch area pacing, Repolarization, Dyssynchronous heart failure

## Purpose

Cardiac resynchronization therapy (CRT) by biventricular pacing (BiVP) reduces symptoms, mortality, and heart failure (HF) hospitalization in patients with dyssynchronous HF. A novel promising CRT approach is left bundle branch area pacing (LBBAP), which presumably generates a more physiological endocardial to epicardial left ventricular activation and thereby potentially a more homogeneous repolarization. The aim of this study was to compare time-dependent repolarization changes in CRT candidates receiving LBBAP vs. BiVP.

## Methods

This was an observational multicentre study adhering to the Declaration of Helsinki and received approval from the institutional review boards of each participating centre, as previously published.^1–5^ Patients eligible for CRT who underwent LBBAP or BiVP in Maastricht University Medical Center (Maastricht, The Netherlands), Policlinico Casilino (Rome, Italy), and Catharina Hospital (Eindhoven, The Netherlands) were included. For the LBBAP cohort, left conduction system capture was confirmed intra-procedurally by observing QRS morphology transitions during threshold testing and achieving a paced V6RWPT of <80 ms or 90 ms (slight variations between centres). Device programming settings were not systematically assessed; however, AV and VV optimization (when relevant) to achieve the shortest paced QRS duration was standard practice. Standard 12-lead electrocardiograms (ECGs) obtained prior to implantation, shortly following implantation, and during 3-, 6-, and 12-month follow-up were analysed using a semi-automatic algorithm.^6^ The assessed repolarization markers included the QT interval as a measure of total action potential duration, the Tpeak-Tend interval as an indicator of repolarization dispersion, and the JT interval as a marker of repolarization duration. All markers were adjusted for heart rate (QTc, JTc, Tpeak-Tend,c) using Fridericia’s formula.

Differences in baseline characteristics were determined using the Mann–Whitney *U* test, Fisher’s exact test, or Fisher–Freeman–Halton exact test where appropriate. Differences in outcome variables within and between groups were determined using mixed models and subsequent Bonferroni’s multiple comparisons tests. Significance was defined as a *P* ≤ 0.05. Categorical data are presented as number of cases (%), and continuous variables are expressed as mean ± standard deviation (SD).

## Results

The study population was representative of a typical CRT cohort, predominantly NYHA class II/III, with an LVEF of 31% ± 7%, 83% LBBB, and a QRS duration of 183 ± 22 ms. Baseline characteristics were not significantly different between the groups, except that BiVP patients were slightly younger than LBBAP patients (67 ± 9.7 vs. 70 ± 8.2 years; *P* = 0.036).

**Figure 1:**
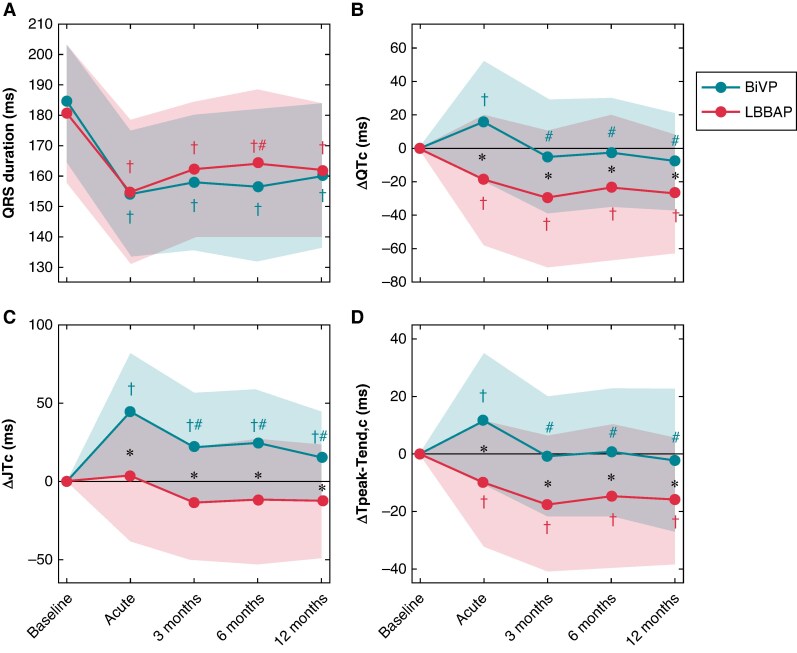
QRS duration (*A*) and changes in QTc (*B*), JTc (*C*), and Tpeak-Tend,c (*D*) compared to baseline following BiVP and LBBAP. BiVP, biventricular pacing; LBBAP, left bundle branch area pacing; ms, milliseconds. *Significant difference between groups. ^†^Significantly different from baseline. ^#^Significantly different from the acute phase. *α* ≤ 0.05.

The results from the ECG analysis are listed in *Table 1*. At baseline, heart rate, QRS duration, and Tpeak-Tend,c were similar for BiVP and LBBAP patients, while QTc and JTc were longer in LBBAP patients compared to BiVP patients (*P* < 0.001). QRS duration (*Figure 1A*) was similar for BiVP and LBBAP patients at all time points and decreased directly and persistently following CRT (*P* < 0.002).

**Table 1:** Results of the ECG analyses

	Baseline	Acute	3 months	6 months	12 months
BiVP (*n* = 95)					
Heart rate (b.p.m.)	68 ± 12	72 ± 12^a^	73 ± 12^a^	68 ± 10^b^	70 ± 11
QRS duration (ms)	185 ± 20	154 ± 21^a^	158 ± 22^a^	156 ± 25^a^	160 ± 24^a^
QTc (ms)	427 ± 33	443 ± 37^a^	420 ± 29^c^	425 ± 29^c^	419 ± 29^c^
JTc (ms)	236 ± 28	280 ± 40^a^	253 ± 32^a,c^	262 ± 33^a,c^	251 ± 27^a,c^
Tpeak-Tend,c (ms)	79 ± 18	90 ± 19^a^	78 ± 14^c^	78 ± 16^c^	75 ± 17^c^
LBBAP (*n* = 57)					
Heart rate (b.p.m.)	65 ± 12	72 ± 14^a^	68 ± 9.5^a,d^	69 ± 11	68 ± 9.4
QRS duration (ms)	180 ± 23	154 ± 24^a^	162 ± 22^a^	164 ± 24^a,c^	162 ± 22^a^
QTc (ms)	447 ± 32^d^	429 ± 31^a,d^	419 ± 32^a^	420 ± 34^a^	421 ± 30^a^
JTc (ms)	263 ± 31^d^	266 ± 34^d^	250 ± 32	250 ± 33^d^	254 ± 33
Tpeak-Tend,c (ms)	84 ± 16	74 ± 18^a,d^	68 ± 20^a,d^	68 ± 19^a,d^	68 ± 15^a,d^

BiVP, biventricular pacing; LBBAP, left bundle branch area pacing; b.p.m., beats per minute; ms, milliseconds; *α* ≤ 0.05.

^a^Significantly different from baseline.

^b^Significantly different from 3 months follow-up.

^c^Significantly different from the acute phase.

^d^Significantly different from BiVP.

The absolute values of QTc, JTc, and Tpeak-Tend,c are listed in *Table 1*. At baseline, QTc and JTc were significantly longer in LBBAP compared to BiVP patients (*P* < 0.001), while during the acute phase, both intervals were longer in BiVP than in LBBAP patients (*P* < 0.025). During follow-up, there were no significant differences in QTc and JTc between groups. Despite having similar Tpeak-Tend,c at baseline, Tpeak-Tend,c was significantly higher for BiVP compared to LBBAP during the acute phase and follow-up (*P* < 0.005).

The time-dependent changes (Δ) in QTc, JTc, and Tpeak-Tend,c are visualized in *Figure 1B–D*. BiVP resulted in an acute prolongation (*P* < 0.001), subsequent reduction towards baseline values (*P* < 0.001), and stabilization afterwards of QTc and Tpeak-Tend,c, while JTc remained prolonged during follow-up (*P* < 0.001). In contrast, LBBAP shortened Tpeak-Tend,c and QTc acutely and permanently (*P* < 0.016 and *P* < 0.009, respectively). Furthermore, ΔQTc, ΔJTc, and ΔTpeak-Tend,c were significantly smaller during LBBAP compared to BiVP at all time points (*P* < 0.006).

## Discussion

This study compared repolarization changes between LBBAP and BiVP in CRT patients at baseline and multiple time points post-implantation. Our data indicate that LBBAP is associated with an acute and permanent decrease in dispersion of repolarization and total action potential duration, while BiVP shows an acute but transient increase in dispersion of repolarization and total action potential duration and permanent increase in repolarization time. Therefore, LBBAP avoids the worsening of repolarization parameters seen acutely after BiVP and generates a more pronounced long-term reverse remodelling of repolarization. This occurs despite a similar degree of activation synchronization. Although QRS duration slightly increased during follow-up in the LBBAP cohort, whether this is due to a change in capture type as a consequence of micro-dislodgement remains to be investigated.

Despite the detrimental changes in repolarization following BiVP, large clinical trials showed no increase in the development of ventricular arrhythmias.^7^ The initial pro-arrhythmic effect of BiVP may be counterbalanced by the long-term anti-arrhythmic effects of CRT through LV reverse remodelling. The seemingly more preferable repolarization changes following LBBAP could further reduce the occurrence of ventricular arrhythmias compared to BiVP, and this concept may explain the reduced occurrence rate of ventricular arrhythmias in LBBAP compared to BiVP as seen in the I-CLAS study.^8^

Potential mechanisms may explain the more favourable repolarization observed with LBBAP compared to BiVP. During BiVP, the area with the shortest action potential duration is activated earlier rather than later,^9^ inducing a larger dispersion of repolarization, which is reflected by the acutely increased QTc, JTc, and Tpeak-Tend,c. The subsequent shortening of these intervals towards or above baseline values are likely a contribution of electrical remodelling.^10^ Following LBBAP, no major changes in repolarization sequence were seen. This seems likely, since the conduction of the action potential through the LV progresses from the LV septum to the LV free wall and from endocardium to epicardium in both baseline LBBB and LBBAP. Furthermore, the fact that Tpeak-Tend,c shortened acutely and did not significantly decrease further in time suggests that the reduction in dispersion of repolarization is a direct consequence of the more synchronous activation of the LV.

## Conclusions

Left bundle branch area pacing was associated with an acute and permanent reduction in dispersion of repolarization and total action potential duration, while BiVP shows a transient increase and no permanent changes. Furthermore, LBBAP does not result in a persistent increase in repolarization time as seen after BiVP. While both LBBAP and BiVP achieve similar levels of activation synchronization, LBBAP demonstrates more favourable repolarization outcomes.

## Data Availability

The data underlying this article will be shared on reasonable request to the corresponding author.
